# Nicotinic Acetylcholine Receptors in HIV: Possible Roles During HAND and Inflammation

**DOI:** 10.1007/s10571-018-0603-8

**Published:** 2018-07-14

**Authors:** Coral M. Capó-Vélez, Manuel Delgado-Vélez, Carlos A. Báez-Pagán, José A. Lasalde-Dominicci

**Affiliations:** 1 Department of Biology, University of Puerto Rico, Río Piedras Campus, PO Box 23360, San Juan, PR 00931 USA; 2 Molecular Sciences Research Center, San Juan, PR 00926 USA; 3 Department of Physical Sciences, University of Puerto Rico, Río Piedras Campus, PO Box 23323, San Juan, PR 00931 USA

**Keywords:** Nicotinic acetylcholine receptor, HIV, Gp120, Inflammation, HAND

## Abstract

Infection with the human immunodeficiency virus (HIV) remains a threat to global health. Since its discovery, many efforts have been directed at understanding the mechanisms and consequences of infection. Although there have been substantial advances since the advent of antiretroviral therapy, there are still complications that significantly compromise the health of infected patients, particularly, chronic inflammation and HIV-associated neurocognitive disorders (HAND). In this review, a new perspective is addressed in the field of HIV, where the alpha7 nicotinic acetylcholine receptor (α7-nAChR) is the protagonist. We comprehensively discuss the available evidence implicating α7-nAChRs in the context of HIV and provide possible explanations about its role in HAND and inflammation in both the central nervous system and the periphery.

## Introduction

Despite the success of the combined antiretroviral therapy (cART), infection with the human immunodeficiency virus type 1 (HIV-1) remains a global health threat. cART has helped prolong the lives of patients, improve their life quality, lessen the occurrence of the acquired immunodeficiency syndrome (AIDS), and decrease AIDS and non-AIDS-related complications; however, it is neither a cure (Spudich 2013) nor is it accessible to every person living with HIV (out of the approximately 36.7 million people infected with the virus, only 18.2 million have access to cART) (Joint United Nations Programme on HIV/AIDS (UNAIDS 2013)).

Besides AIDS, HIV infection results in a series of systematic physiological complications such as chronic inflammation and HIV-associated neurocognitive disorders (HAND), regardless of cART treatment (Appay and Sauce 2008; Heaton et al. 2011; Harezlak et al. 2011; Funderburg et al. 2014). These complications do not receive sufficient attention and continue to be a serious threat to the quality of life of patients living with the virus (Saylor et al. 2016; Althoff et al. 2016). Sadly, the mechanisms leading to either of these complications are not completely understood and no specific treatment strategies are available to this date.

Taking this into account, numerous efforts have aimed to better understand the mechanisms used by the virus during infection in order to identify new therapeutic targets. Specifically, several studies have presented evidence implicating ion channels in HIV infection (Raber et al. 1996; Holden et al. 1999; Gelman et al. 2004; Herman et al. 2010; Chen et al. 2011; Liu et al. 2013; Tewari et al. 2015), suggesting that they could be used as potential pharmacological targets. Among these ion channels, nicotinic acetylcholine receptors (nAChRs) have been suggested to play important roles (Bracci et al. 1992; Giunta et al. 2004; González-Lira et al. 2006; Rock et al. 2008; Ballester et al. 2012; Cao et al. 2013; Gundavarapu et al. 2013; Zhang et al. 2015; Báez-Pagán et al. 2015; Ramos et al. 2015; Delgado-Vélez et al. 2015; Liu et al. 2017; Ekins et al. 2017; Capó-Vélez et al. 2018); however, these have not yet been extensively explored. In particular, the α7-nAChR is emerging as an important player in the HIV field, since it is expressed not only in the brain, but also in a wide variety of immune cells that are targeted during HIV infection, such as macrophages, monocytes, B-lymphocytes, and T-lymphocytes (CD4^+^) (Wang et al. 2003; van der Zanden et al. 2012; Kawashima et al. 2015), making it a suitable target for treatment development.

Due to the presence of α7-nAChR on both neuronal and immune cells, and its known participation in HIV settings, this review will focus on the role of α7-nAChRs in two different but equally important aspects of HIV: inflammation (neuroinflammation and peripheral inflammation) and HAND, both of which severely compromise the quality of life of patients infected with HIV. Here, we review the role of α7-nAChRs in these HIV-derived consequences and suggest the receptor as a possible pharmacological target to treat/ameliorate both HAND and inflammation.

## HIV, cART, and HAND: the Current Status

HIV infection continues to be a major global public health issue, having claimed more than 35 million lives so far (UNAIDS 2017). The currently available treatment, i.e., cART, has substantially decreased AIDS-related hospital admissions (Center for Disease Control and Prevention 2014) and changed the course of HIV infection from a defining disease to a manageable condition. Consequently, treating patients with cART declined AIDS-related deaths, decreased maternal-infant viral transmission, and decreased the incidence of HIV-associated dementia (HAD) (Maschke et al. 2000; Saylor et al. 2016).

Although cART is greatly beneficial (INSIGHT START Study Group et al. 2015), it has somewhat limited therapeutic results including long-term toxicity, dependence on patient’s daily adherence, and drug–drug interactions, among others (Solomon and Sax 2015). In fact, despite the decrease in viral load and improved or normal CD4^+^ T-cell counts (Antinori et al. 2007), patients still suffer from chronic inflammation (Appay and Sauce 2008; Funderburg et al. 2014); thus, the need for new and innovative therapies is clearly of the utmost importance.

Inflammation is a defense mechanism against diverse insults, and it is programmed to eliminate or remove exogenous noxious agents. This process is mediated by effector molecules such as cytokines, which are released by immune cells during immune responses. Cytokines are key to innate immune responses; however, excessive cytokine production can give rise to chronic or uncontrolled inflammatory responses, an event related to the pathology of many inflammatory diseases (Johnston and Webster 2009; Wang et al. 2009). Inflammation is controlled through the cholinergic anti-inflammatory pathway (CAP), which seems to be altered in HIV patients (Delgado-Vélez et al. 2015). In non-infected patients, impairment or disruption of the CAP is associated with delirium, depression, ulcerative colitis, postoperative cognitive decline, inflammatory bowel disease, hemorrhagic shock, pancreatitis, exacerbation of ventilator-induced lung injury, septic peritonitis, hypotensive shock, and cardiopulmonary arrest (Lindgren et al. 1993; Borovikova et al. 2000; van Westerloo et al. 2005, 2006; Das 2007; Tracey 2009; dos Santos et al. 2011; Norman et al. 2011, Su et al. 2012; Cerejeira et al. 2012).

Furthermore, around 15–50% of infected individuals develop some form of cognitive and/or motor dysfunctions, collectively known as HAND (Heaton et al. 2010, 2011; Harezlak et al. 2011; Spudich 2013; McGuire et al. 2014; Saylor et al. 2016). It is a frequent complication of HIV infection that causes several neurological disorders including dementia, myelopathy, peripheral neuropathy, and myopathy (Price and Perry 1994; Lindl et al. 2010). HAND is a heterogeneous complication of HIV, being HAD the most severe form of neurological alteration. At one time, HAD was estimated to affect 20–30% of infected patients, but after therapies became available and cART was introduced, this percentage decreased significantly (Heaton et al. 2011). cART treatment predominantly shifted HAND neurological manifestations to milder forms of cognitive impairment instead of the frank dementia seen in the pre-cART era (Antinori et al. 2007). Although antiretroviral therapy has been helpful in the treatment of HAD, there is a therapeutic gap within the milder form of the disease, where cART is less effective (McGuire et al. 2014). The milder forms of HAND, minor cognitive dysfunction (MCD), and asymptomatic neurocognitive impairments (ANI), are estimated to affect around 15–50% of HIV^+^ patients (Simioni et al. 2010; Heaton et al. 2010; Harezlak et al. 2011; Tavazzi et al. 2014; Saylor et al. 2016). This highlights the need to develop innovative pharmacological strategies because therapies aimed to decrease or abrogate HAND cannot necessarily depend on drugs that decrease HIV replication.

The brain is one of the organs most affected by HIV infection (Woods et al. 2009). This reflects the fact that several cells present in the brain, mainly microglia, can be actively infected by HIV since they express receptors needed for infection (Price and Perry 1994). HIV does not infect neurons; however, HAND patients experience a remarkable loss of neurons due to interaction with viral particles, which trigger the release of inflammatory cytokines (Kaul et al. 2001). Neuronal injury is believed to result from a direct effect and/or interaction between viral proteins and the cell, and indirect effects, which are mediated by activated macrophages that secrete neurotoxic factors such as TNF-α, IL-6, and IL-1β. These neurotoxic factors and/or cytokines can interact with cell surface receptors in neurons and/or astrocytes and activate signaling pathways that could lead to apoptosis (Saylor et al. 2016).

Thus, HAND is an important cause of cognitive dysfunction and, together with chronic inflammation, remains an important unresolved issue stemming from HIV. Both are equally important aspects that require urgent attention to counteract their chronic deleterious effects.

## Nicotinic Acetylcholine Receptors

Nicotinic acetylcholine receptors are fast ionotropic ion channel receptors that bind to and are activated by their endogenous ligands acetylcholine (ACh) and choline; they also bind nicotine (Gahring and Rogers 2005). Neuronal nAChRs include α (α2–α10) and β subunits (β2–β4) arranged in different stoichiometries to form functional receptors (e.g., α4β2, α2β2, α2β4, α3β2, and α4β4, among others). These heteromeric receptors (assembled with more than one type of subunit) are characteristic for their high affinity to nicotine but differ in their pharmacological properties depending on subunit composition (Gahring and Rogers 2005).

The nAChR subunits α2, α3, α4, α10, β2, and β4 are not able to form functional ion channels by themselves. Conversely, subunits α7–α9 form homomeric receptors (assembled with only one type of subunit) and comprise a family of receptors that bind nicotine with lower affinity than heteromeric receptors but bind the antagonist snake toxin, α-bungarotoxin (α-bgtx), with high affinity (Breese et al. 1997; Rangwala et al. 1997). Moreover, studies have shown that nAChRs can modulate cellular processes in the central nervous system (CNS). For instance, nicotine administration can improve cognitive performance in normal subjects and produce a number of physiological effects (Ochoa and Lasalde-Dominicci 2007).

Studies performed by Bracci et al. demonstrated a significant homology between a specific sequence of gp120, the coat protein of HIV, and the putative active sites of snake curare-mimetic neurotoxins, which have the ability to bind irreversibly to nAChRs. Furthermore, the authors demonstrated that recombinant gp120 inhibits the binding of the nAChR antagonist, α-bgtx, suggesting that other type of receptors (such as nAChRs) can function as HIV receptors, and supports the notion that ion channels may have a role during HIV infection. Even though this study suggested a potential role for nAChRs in HIV, no specific nAChR subtype was implicated (Bracci et al. 1992). Subsequent studies performed in SIV-infected monkeys found that reduced cholinergic neurotransmission was present in the form of a dramatic reduction in choline acetyltransferase activity, the enzyme responsible for the biosynthesis of ACh (Koutsilieri et al. 2000). Consistent with these observations, González-Lira et al. (2006) performed electrophysiological studies in rats and found that, in addition to motor and cognition impairments, gp120 interferes with cholinergic neurotransmission as part of the neuropsychiatric abnormalities characteristic of HAD. However, nicotine administration maintained these parameters near normality, supporting the participation of nAChRs in the beneficial outcome. An interesting proof-of-concept study was designed and carried out to evaluate if an increase in the bioavailability of ACh, through the chemical inhibition of acetylcholinesterase, was capable of enhancing the anti-inflammatory reflex in patients infected with HIV (Valdés-Ferrer et al. 2009). Results demonstrate that pyridostigmine (an acetylcholinesterase inhibitor) was able to modify the overactivation and proliferation of T-lymphocytes in patients chronically infected with HIV. This approach also led to an increase in the anti-inflammatory cytokine IL-10, a decrease in T-lymphocytes proliferation, and the production of the proinflammatory cytokine IFN-γ. Moreover, recently, a 16-week proof-of-concept open trial was performed using pyridostigmine as add-on therapy in seven HIV-infected patients with discordant immune response receiving combined antiretroviral therapy, to determine whether pyridostigmine would promote an increase in total CD4^+^ T-cells (Valdés-Ferrer et al. 2017). Results indicate that in patients suffering from HIV^+^, add-on pyridostigmine results in a significant and persistent increase in circulating CD4^+^ T-cells.

Altogether, these studies suggested a role for nAChRs in HIV. However, considering the pharmacological properties and different roles of the α7-nAChR, a closer look is justified. Below we describe the proposed functions/roles of this receptor to then highlight studies specifically relating to HIV-induced inflammation and HAND, where α7-nAChRs play a critical part.

## The α7-nAChR: A Unique Ion Channel with Multiple Roles

Many studies have identified roles in health and disease for the α7-nAChR (Ochoa and Lasalde-Dominicci 2007; Counts et al. 2007; Duffy et al. 2009). The α7-nAChR is a ligand-gated ion channel that is rapidly desensitized in the presence of a ligand (Conejero-Goldberg et al. 2008). This neuronal receptor is found predominantly in the cerebral cortex, thalamus, hippocampus, basal ganglia, and hypothalamus (both pre- and post-synaptically) (Vázquez-Palacios and Bonilla-Jaime 2004), and immune cells (Wang et al. 2003; Yoshikawa et al. 2006; van der Zanden et al. 2012; Kawashima et al. 2015). Furthermore, α7-nAChRs can be localized in the soma, presynaptic terminals (regulating neurotransmitter release), and postsynaptic terminals (modulating neuronal excitability) (Gahring and Rogers 2005). Once activated, its ion pore opens to permit the flow of Na^+^, K^+^, and particularly Ca^2+^.

The mechanisms by which the α7-nAChR modulates neurotransmitter release and neuronal excitability are distinct from those proposed for other nAChRs and reflect the channel’s unique pharmacological properties. For example, the α7-nAChR binds α-bgtx with high affinity (Kd = 94 pM) (Rangwala et al. 1997) and has low affinity for agonists such as nicotine (Mansvelder et al. 2009). Furthermore, at low agonist concentrations, activation of α7-nAChR induces a Ca^2+^ influx, leading to a Ca^2+^-induced Ca^2+^ release from intracellular stores (Mansvelder et al. 2009).

In general, the proposed function for α7-nAChRs in the brain is to modify the excitability of neurons (Paterson and Nordberg 2000). For example, α7-nAChRs are expressed in the striatum, where they play a role in the function of local neuronal circuits (Martin 2003). A study conducted by Wu et al. (2004) showed that α7-nAChRs are expressed on neurons from the substantia nigra and the ventral tegmental area and demonstrated that, once activated, α7-nAChRs can modulate dopamine release. These results suggest an involvement of the α7-nAChR in dopaminergic neuronal function and drug reinforcement mechanisms, particularly nicotine dependence. Moreover, nicotine stimulates dopamine release in the nucleus accumbens; an effect thought to be mediated by an increase of glutamate release by α7-nAChRs located on striatal glutamatergic neurons. This stimulates the release of dopamine in the nucleus accumbens (Kaiser and Wonnacott 2000), further supporting the proposed role of α7-nAChRs as modulators of neurotransmitter release in the CNS. Therefore, α7-nAChRs can modulate various signaling pathways that converge to fine-tune neuronal circuits and contribute to subtle but crucial aspects of cognitive behavior (Mansvelder et al. 2009). Consequently, the expression of small numbers of α7-nAChRs at regulatory sites may lead to multiple outcomes in the performance of normal cells, susceptibility to exogenous agents, or participation in processes ranging from neurodegeneration to inflammation (Gahring and Rogers 2005).

**Fig. 1:**
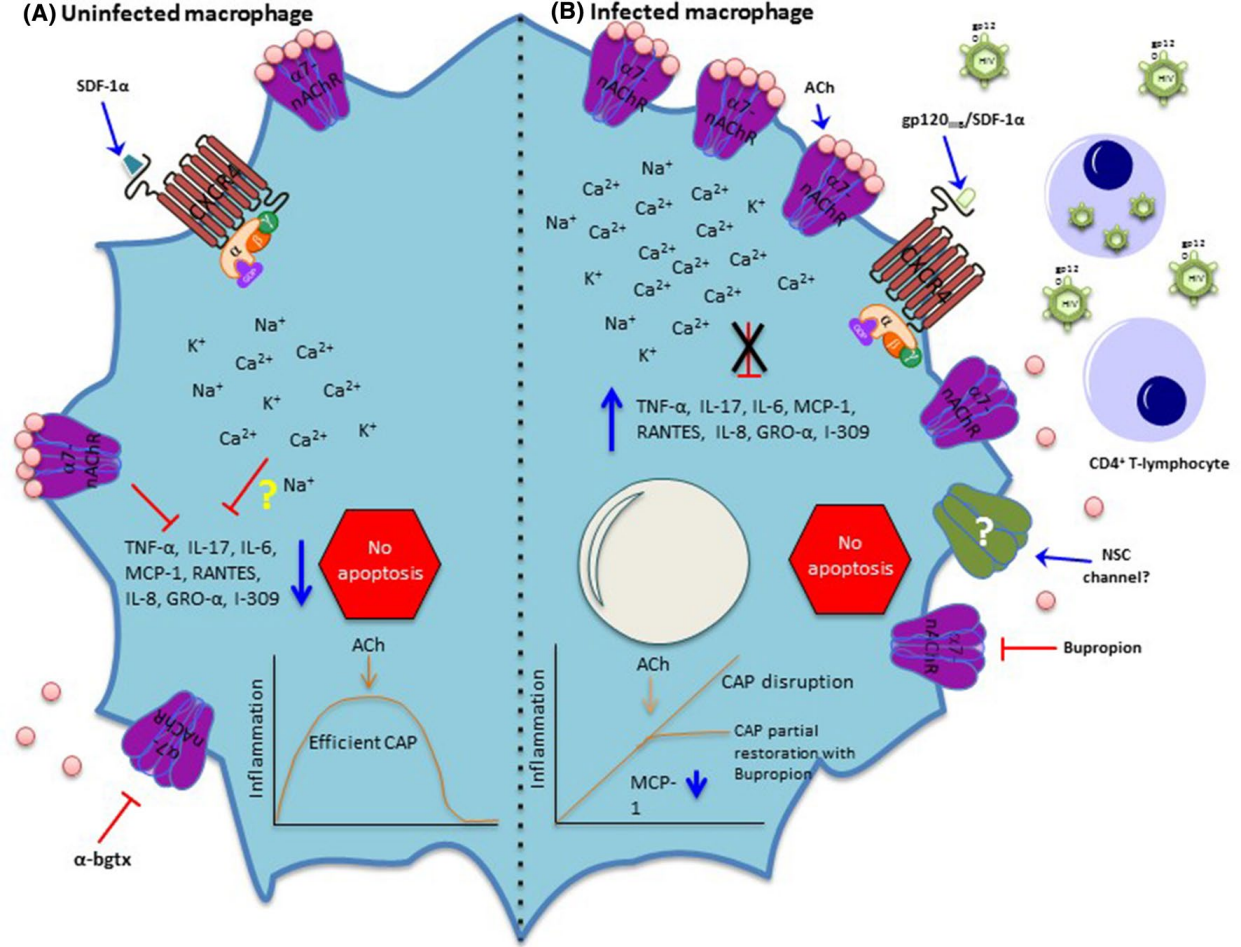
Effects of HIV infection on macrophages’ α7-nAChRs. **a** In uninfected macrophages, SDF-1α promotes signaling through CXCR4, a G-protein coupled receptor. In addition, as mentioned before, α7-nAChR is sensitive to the antagonist α-bgtx. A robust body of evidence demonstrates that the net effect of α7-nAChR activation is inhibition of the production of cytokines (Wang et al. 2003), consequently decreasing inflammation (efficient CAP). Whether the ion influx through α7-nAChR is relevant for the proper operation of the CAP or other important physiological processes remains obscure. **b** In infected macrophages, HIV-infected CD4^+^ T-lymphocytes secrete virions and gp120_IIIB_ that interact and stimulate CXCR4 to induce the upregulation of α7-nAChR (Delgado-Vélez et al. 2015). This upregulation does not result in cell death, or apoptosis, which is consistent with the anti-apoptotic signature expressed by monocytes recovered from patients infected with HIV (Giri et al. 2009) and those from HIV-infected macrophages (Swingler et al. 2007). Contrary to what was expected, the activation of this high number of α7-nAChRs with ACh does not enhance the CAP response because their activation does not lead to inhibition of cytokine production, therefore, conferring macrophages a proinflammatory phenotype indicative of a CAP disruption. Moreover, application of an antagonist of α7-nAChR, bupropion, partially restores the CAP by reducing inflammatory chemokines, but not interleukins (Delgado-Vélez et al. 2015). Interestingly, whether the identity of the non-selective calcium channel previously described (Liu et al. 2000) is α7-nAChR remains to be determined

In addition to its central role in the CNS, the α7-nAChR is also present in immune cells. As a result, several efforts have been made to elucidate its role in these cells. It is now clear that the α7-nAChR is an essential regulator of inflammation in macrophages through the CAP (Tracey 2007). The anti-inflammatory activity of the α7-nAChR occurs by the ACh-induced activation of the receptor to inhibit the production of proinflammatory cytokines in macrophages, thereby modulating immune responses and the progression of inflammatory diseases, avoiding organ and systemic damage (Tracey 2009). Altogether, these studies highlight the importance of this neuroimmune tract for the well-being of healthy people and accents the α7-nAChR’s pivotal role in the innate immune response necessary to resolve inflammation. Despite its well-known role in the CAP, the lack of functional (electrophysiological) evidence has raised the question of whether this receptor acts as an ion-conducting channel (Skok 2009). However, we recently demonstrated that, in human monocyte-derived macrophages (MDMs), the α7-nAChR retains its ion channel translocation activity (Báez-Pagán et al. 2015). Whether translocated ions play a role in the CAP remains largely unknown (Fig. 1).

Even though the anti-inflammatory role of α7-nAChR in monocytes and macrophages is well recognized (Wang et al. 2003; Yoshikawa et al. 2006), its role in other immune cells is not well understood. Recent reports demonstrate that α7-nAChR plays an essential role in the immune synapse between human T- and B-lymphocytes since they are recruited to these sites to inhibit CD40-related mitogenic functions (Kalashnyk et al. 2012). It also regulates B-lymphocytes’ proliferation (Koval et al. 2009), development, and survival (Skok et al. 2006). Moreover, a recent study identified the α7-nAChR as a modulator of lymphocyte activation (De Rosa et al. 2009). In the case of regulatory T-lymphocytes (Tregs), the α7-nAChR seems to be a critical regulator of the immunosuppressive function of CD4^+^ CD25^+^ Tregs (Wang et al. 2010). However, it is also important to mention that, in addition to α7-nAChRs, monocytes, macrophages, B-lymphocytes, and T-lymphocytes also express other homomeric nAChRs, including α3, α5, α9, and α10 (Wang et al. 2003; Kawashima et al. 2012), receptors that could contribute to the net cellular response of these cells during health and disease.

As a whole, this demonstrates the importance of α7-nAChRs in various cellular processes and highlights its versatility. Thus, this receptor is an integral player of innate processes ranging from the brain to immune cells having clear repercussions in neurotransmitter release, neurodegenerative diseases, and inflammation.

## HIV-Induced Inflammation/Neuroinflammation and α7-nAChR

The general notion is that in patients infected with HIV, the inflammatory response is exaggerated, uncontrolled, and chronic. Undoubtedly, patients infected with HIV suffer from chronic and persistent inflammatory processes, which cause numerous AIDS and non-AIDS-related complications such as neurocognitive deterioration, cardiovascular disease, thromboembolic disease, type II diabetes, cancer, osteoporosis, multiple end-organ disease, and frailty (Deeks et al. 2013; Barré-Sinoussi et al. 2013; Hearps et al. 2014), even when patients adhere to cART regimens that minimize viral replication and increase CD4 cell counts (Appay and Sauce 2008; Funderburg et al. 2014).

In the CNS, the relationship between neuroinflammation and α7-nAChRs in the context of HIV has been scarcely examined. Available evidence suggests that activation of α7-nAChRs expressed by microglia (brain macrophages) plays a key role during neuroinflammation processes since its activation seems to suppress inflammation through the CAP, the same mechanism used by macrophages, thus conferring neuroprotective properties (Shytle et al. 2004; Suzuki et al. 2006). Giunta et al. (2004) reported an in vitro model of HIV-associated dementia composed of cultured microglia cells synergistically activated by IFN-γ and gp120. However, this activation was synergistically attenuated through α7-nAChRs and the p44/42 MAPK pathway by pretreatment with nicotine and the cholinesterase inhibitor, galantamine.

The detrimental consequences of HIV infection are not limited to the presence of viral constituents in the body, such as gp120. Accumulating evidence also demonstrates that neuroinflammation could emerge from soluble virions, HIV-infected monocytes, and T-lymphocytes that cross the permeabilized blood–brain barrier (BBB). These infected cells not only affect brain-resident cells upon migration into the CNS but also produce proinflammatory cytokines such as TNF-α and IL-1β, which in turn, activate microglia and astrocytes. Cytokine levels in the cerebrospinal fluid are associated with neurocognitive impairment in HIV infection. Activated brain-resident cells (e.g., microglia), along with perivascular macrophages, are the main contributors to neuroinflammation in HIV infection (Hong and Banks 2015). They can release neurotoxic factors, such as excitatory amino acids and inflammatory mediators, resulting in neuronal dysfunction and death (Hong and Banks 2015). Moreover, viral proteins (e.g., gp120), which induce brain endothelial cells to release cytokines, may also contribute to brain inflammation and the pathogenesis of HAND. In fact, gp120 facilitates infiltration of HIV-infected immune cells due to permeabilization of the BBB (Kanmogne et al. 2007). Interestingly, a recent study was conducted to evaluate the effects of antiretroviral therapy on the brain. Results showed that indinavir (a protease inhibitor) acts as a positive allosteric modulator of α7-nAChRs at low concentrations, whereas at high concentrations, it acts as an inhibitor of α7-nAChRs in the brain, thus possibly contributing to the cognitive dysfunction observed in patients infected with HIV (Ekins et al. 2017). However, caution is needed regarding the possible effects of antiretroviral therapy on the CNS.

Another report on this matter demonstrated that the α7-nAChR agonists, choline, and GTS-21 attenuated the intrathecal gp120-induced proinflammatory cytokine- and microglia-dependent mechanical allodynia (Loram et al. 2010). Of note, choline (full agonist of α7-nAChR) significantly blocked and reversed the gp120-induced mechanical allodynia for at least 4 h after administration. In addition, intrathecal choline delivered either with or 30 min after gp120 reduced the gp120-induced production of IL-1β protein and proinflammatory cytokine mRNAs within the lumbar spinal cord. The second α7-nAChR agonist, GTS-21, also significantly reversed the gp120-induced mechanical allodynia and lumbar spinal cord levels of proinflammatory cytokine mRNAs and IL-1β protein.

The CAP is an innate neuroimmune mechanism whereby cytokine production is inhibited via activation of α7-nAChRs expressed in macrophages; this avoids tissue and organ damage due to an exaggerated and persistent inflammation (Tracey 2002; Wang et al. 2009). This pathway provides a fast, discrete, and localized way of controlling immune responses and regulating inflammation (Johnston and Webster 2009) triggered by proinflammatory molecules (e.g., cytokines, LPS). Upon its detection, a signal travels to the CNS via the afferent vagus nerve fibers and stimulates the release of ACh by the efferent arm of the vagus nerve. Then, ACh activates α7-nAChRs in macrophages to inhibit cytokine production and balance the inflammatory response (Tracey 2002, 2009; Wang et al. 2009). In addition, electrical stimulation of the vagus nerve and α7-nAChR agonists ameliorate symptoms in models of inflammatory diseases, demonstrating a pivotal role for α7-nAChRs in the regulation of inflammation.

Interestingly, this mechanism is conserved in microglia, where α7-nAChRs regulate neuroinflammation (Shytle et al. 2004). Because both macrophages and microglia regulate inflammation through the same mechanism, it could be speculated that they could regulate each other. Indeed, a recent study demonstrates that electrical activation of the vagus nerve decreases the levels of proinflammatory cytokines and the percent of activated microglia in the brain (Meneses et al. 2016). These results provide an important piece of information since neuroinflammation is a hallmark of several infections and neurodegenerative diseases. Thus, strategies to control neuroinflammation could result in a definite improvement in patients (Meneses et al. 2016).

Although the importance of the CAP is evident, so far it has not been studied extensively in patients infected with HIV. Recently, our group examined the cholinergic anti-inflammatory response (CAR) in HIV. We found that gp120_IIIB_ addition to MDMs induces upregulation of α7-nAChRs in vitro (Fig. 1). Moreover, inflammation studies performed in MDMs pre-exposed to gp120_IIIB_ and challenged with LPS demonstrated that ACh addition is no longer able to reduce inflammation, suggesting a CAR disruption. Furthermore, examination of α7-nAChR levels in monocytes, MDMs, and T-lymphocytes recovered from patients infected with HIV revealed a remarkable upregulation in these cell types (Delgado-Vélez et al. 2015). Finally, application of bupropion (an α7-nAChR antagonist) to MDMs upregulated for α7-nAChR (by gp120) partially restores the CAR by reducing inflammatory chemokines, but not interleukins (Delgado-Vélez et al. 2015) (Fig. 1).

Overall, HIV-induced inflammation occurs in two scenarios: the periphery and the CNS. Nevertheless, the specific mechanisms by which they occur are not entirely understood, and further studies are needed to identify new therapeutic targets to generate new neuroprotective medications. The studies described here highlight the importance of considering the α7-nAChR and the CAP in HIV-induced pathologies and expand the current knowledge about neuroimmunomodulation processes during HIV infection.

## HAND and the α7-nAChR

The α7-nAChR is amply distributed through the brain. In a series of works, the α7-nAChR has been implicated in different HIV-related settings. It was shown that gp120 injected into the hippocampus impairs memory by affecting the cholinergic/vasoactive intestinal peptide system (Farr et al. 2002). More recently, a report implicated α7-nAChRs in Cryptococcal-induced meningitis, the most common fungal opportunistic infection in the CNS of patients infected with HIV(Zhang et al. 2015). Specifically, the authors demonstrated that α7-nAChRs play a detrimental role in the host’s defense against *Cryptococcus neoformans* and HIV-1 associated comorbidity factors that compromise BBB integrity (Zhang et al. 2015). Furthermore, a recent report suggests that α7-nAChR may play an important role in the neuropathology caused by gp120, methamphetamine, and nicotine, which are the major pathogenic factors contributing to the pathogenesis of HAND (Liu et al. 2017). Moreover, Gundavarapu et al. (2013) demonstrated that HIV-gp120 induces and regulates mucus formation on airway epithelial cells through a CXCR4-α7-nAChR-GABA_A_R-dependent pathway.

**Fig. 2:**
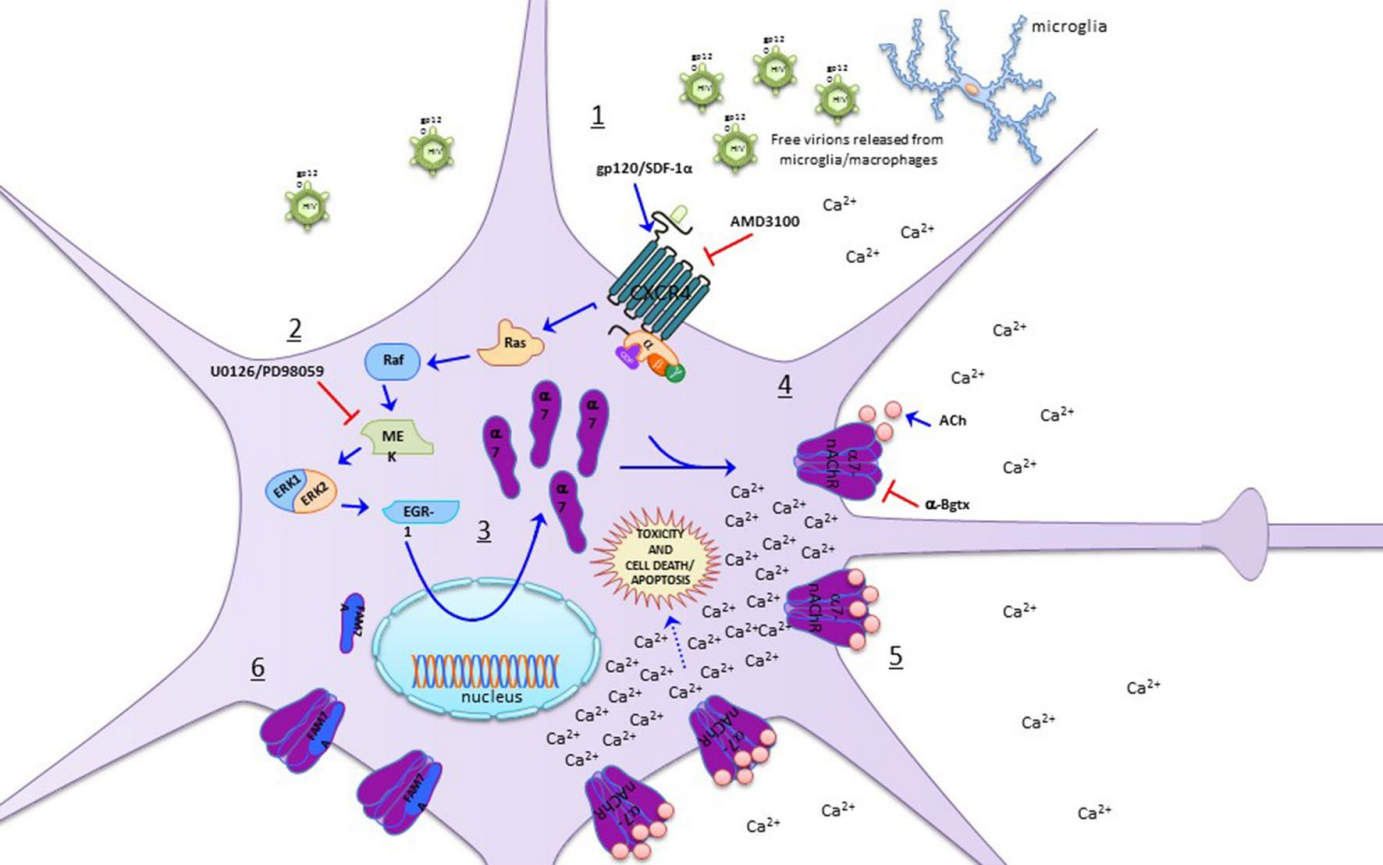
Effects of HIV infection on neurons’ α7-nAChRs. *1*–*4* gp120 released from HIV-infected microglia interact with CXCR4 expressed by neurons, activating the Ras/Raf/MEK/Erk/Egr-1 signaling pathway that eventually promotes α7-nAChR upregulation in neuronal cells (Ballester et al. 2012). Moreover, this response can be recapitulated by adding SDF-1α, the endogenous agonist of CXCR4, which also induces α7-nAChR overexpression in neuronal cells (Ballester et al. 2012). These responses are sensitive to CXCR4 blockade with AMD3100 and MEK inhibition by U0126 or PD98059. *5* Furthermore, α7-nAChR activation with ACh promotes intracellular Ca^2+^ overload that results in cell death and apoptosis (Ballester et al. 2012; Capó-Vélez et al. 2018). *6* The gp120-induced α7-nAChR upregulation described in points *1*–*5* involves a decrease in dupα7 expression, its repressive partial duplication (Ramos et al. 2015)

In Ballester et al. (2012), a potential role for α7-nAChR in HAND was established, as the HIV-1 coat protein gp120_IIIB_ was shown to induce a functional upregulation of α7-nAChRs promoting an increase in Ca^2+^ entry that resulted in neuronal cell death (Fig. 2). Along this line, Ramos et al. (2015) showed that, in neuronal cells, gp120_IIIB_ is able to increase the expression of the α7-nAChR gene, *CHRNA7*, together with a down-regulation of *CHRFAM7A*, the partial duplication of the α7-nAChR gene (dupα7) (Fig. 2). Moreover, evaluation of these genes in the basal ganglia of patients infected with HIV suffering from different stages of HAND demonstrates an alteration in the *CHRFAM7A*/*CHRNA7* expression ratio. Finally, Capó-Vélez et al. (2018) demonstrated that, in mice expressing gp120_IIIB_ in the brain, striatal neurons are upregulated for α7-nAChRs, and its activation leads to an increase in intracellular calcium and eventual apoptosis, an effect that can be attenuated by antagonizing the α7-nAChR (Fig. 2). Moreover, they showed that gp120_IIIB_ mice perform worse than wild-type mice on a striatum-dependent behavioral task and demonstrate locomotor impairments, which improve significantly with α7-nAChR antagonists (Capó-Vélez et al. 2018).

Taken together, the available literature (summarized in Table 1) highlights the importance of studying the role of the α7-nAChR in an HIV-related setting and demonstrates its feasibility as a pharmacological target.

**Table 1:** Studies that establish relationships between HIV and α7-nAChR

nAChR or subtype	Finding(s)	Relevance to HIV field	Reference
nAChRs	A significant homology between HIV-gp120 and snake curare-mimetic neurotoxins, which specifically bind to nAChRs was found	The first suggestion was that HIV-1 gp120 could bind to nAChR expressed in muscle cells and neurons. In addition, the nAChR was proposed as an HIV receptor	
	Nicotine, a nAChR agonist, enhances production of HIV of in vitro-infected alveolar macrophages from healthy cigarette smokers	HIV-infected smokers should experience an increase in viral load	
	HIV-gp120 injected in the hippocampus impairs memory in rats, an effect reversed by hippocampal cholinergic stimulation	Cholinergic pathways could have an essential role in reversing the cognitive decline induced by gp120 neurotoxicity	Farr et al. (2002)
	The coat protein gp120 is involved in the pathogenesis of neuropsychiatric abnormalities; it occurs by interfering with cholinergic neurotransmission	Propose a mechanism of action for gp120 by interfering with cholinergic pathways, a phenomenon that could be part of the etiology of cognitive disturbances. Importantly, nicotine treatment seems to be beneficial	González-Lira et al. (2006)
	Nicotine-treated microglia show increased HIV-1 expression in a concentration-dependent manner	nAChRs are important in the neuropathogenesis of CNS in patients infected with HIV	Rock et al. 9^2008^)
	Nicotine treatment restored gp120-impaired pathways in the brain of HIV-transgenic rats including the prefrontal cortex, dorsal hippocampus, and dorsal striatum	Nicotine has beneficial effects on HIV-induced neurological deficits, presumably through the activation of nAChRs in the brain	Cao et al. (2013)
α7-nAChRs	α7-nAChR is expressed in macrophages, where it is an essential regulator of inflammation acting through the cholinergic anti-inflammatory pathway	Since chronic inflammation is a common phenomenon in patients infected with HIV (despite good viral control and CD4^+^ recovery), the α7-nAChR could be an important target for the regulation of inflammation	Wang et al. (2003)
	In cultured microglia activated with both interferon and gp120 (an in vitro model of HAD), microglial activation was attenuated by pretreatment with nicotine and galantamine. This occurred through an α7-nAChR and p44/42 MAPK-dependent mechanism	Novel therapeutic combination to treat or prevent HAD development through modulation of microglial activation using α7-nAChR agonists and/or acetylcholinesterase inhibitors	Giunta et al. (2004)
	Choline, an α7-nAChR-specific agonist (Alkondon et al. 1997), significantly blocked and reversed gp120-induced mechanical allodynia	α7-nAChR as a potential target to modulate and/or abrogate gp120-induced allodynia	Loram et al. (2010)
	Exposure of neuronal cells to gp120 induces a functional α7-nAChR upregulation that leads to an increase in calcium influx and subsequent cell death. The α7-nAChR upregulation was also observed in a gp120-transgenic mouse model, specifically in the striatum (basal ganglia), a region greatly affected in patients infected with HIV	Suggest a novel pathway for gp120-induced neuronal cell death Results position the α7-nAChR as a potential therapeutic target to ameliorate or reverse HAND-related cognitive dysfunction since antagonists for this receptor are commercially available	Ballester et al. (2012)
	Normal human broncoepithelial cells produce mucus in response to CXCR4-tropic gp120. Mucus formation was blocked by CXCR4, α7-nAChR, and GABA_A_R inhibitors	First demonstration that HIV-gp120 induces and regulates mucus formation in the airway epithelial cells through the CXCR4-α7-GABA_A_R pathway, which may contribute to the higher incidence of obstructive pulmonary diseases in this population	Gundavarapu et al. (2013)
	In neuronal cells where the α7-nAChR is upregulated after gp120 exposure, measurements of α7-nAChR expression gene (*CHRNA7)* and its partial duplication, dupα7, (*CHRFAM7A)* reveal that the *CHRNA7* increase is accompanied by a reduction of its repressive partial duplication, *CHRFAM7A*. Moreover, there is a dysregulation of evaluation of *CHRNA7* and *CHRFAM7A* expression in postmortem brain samples from patients infected with HIV suffering from different stages of HAD	Showed dysregulation in *CHRFAM7A*/*CHRNA7* expression ratio in the basal ganglia from postmortem brain samples of HIV-infected subjects. These results coupled with the high calcium permeability of α7-nAChR may explain the significant loss of neurons in this area, which is a common manifestation of HIV-induced neurological disorders (Everall et al. 1995; Brew et al. 1995)	Ramos et al. (2015)
	Both chemical and genetic blockage of α7-nAChRs are protective against *C. neoformans*- and HIV-1 associated comorbidity factors-induced BBB injury and CNS disorders by down-regulation of circulating brain microvascular endothelial cells (cBMEC) shedding, monocyte recruitment, NF-κB signaling, senescence, and neuronal inflammation	Pharmacological blockade of α7-nAChR in the BBB could prevent HIV-1 associated comorbidity factors-induced BBB injury and CNS disorders	Zhang et al. (2015)
	The HIV Env gp120 induces the upregulation of α7-nAChR in MDMs. In addition, patients infected with HIV are upregulated for α7-nAChR in their monocytes, MDMs, and T-lymphocytes. Activation of the cholinergic anti-inflammatory response in α7-nAChR-upregulated MDMs shows that gp120 disrupts this innate immune mechanism to control inflammation. Furthermore, application of an α7-nAChR antagonist, bupropion, partially restores the response	α7-nAChR emerges as a potential pharmacological target to control chronic inflammation in HIV-infected subjects	Delgado-Vélez et al. (2015)
	They demonstrate that blockade of α7-nAChR could significantly reduce HIV-1 gp120, methamphetamine, and nicotine-induced BBB injury and CNS disorders by decreasing amyloid-beta transport, leukocyte recruitment, cholinergic signaling, premature senescence of brain microvascular endothelial cells, and neuroinflammation	This work suggests that α7-nAChR may play an essential role in neuropathology caused by gp120, methamphetamine, and nicotine, which are the major pathogenic factors contributing to the pathogenesis of HAND	Liu et al. (2017)
	This group demonstrated that at low concentrations, indinavir (a protease inhibitor), acts as a positive allosteric modulator of α7-nAChR, whereas at concentrations greater than 10 mmol/l indinavir acts as an inhibitor of the α7-nAChR	This work demonstrates that an antiretroviral therapeutic drug interferes with the α7-nAChR in the brain, thus possibly contributing to the cognitive dysfunction observed in patients infected with HIV. Moreover, it opens a venue to investigate the effects of other antiretrovirals on α7-nAChR and expand the efforts to other cholinergic receptors involved in cognition	Ekins et al. (2017)
	In a murine model of gp120_IIIB_ expressed in the brain, it was found that α7-nAChR is overexpressed in striatal neurons and its activation promotes apoptosis. Moreover, a striatum-dependent task showed that these animals have a poorer performance and exhibit locomotor impairment. Furthermore, administration of α7-nAChR antagonists improved the locomotor performance in these animals significantly	Consistent with Zhang et al. (2015) and Delgado-Vélez et al. (2015), results of this work position α7-nAChR as an attractive pharmacological target that should be exploited in the HIV field	Capó-Vélez et al. (2018)

## Concluding Remarks

HIV infection represents a major burden to the immune system. Decades of basic and clinical research have made it clear that HIV infection promotes uncontrolled inflammation in both the periphery and the CNS of infected subjects. Furthermore, HIV infection does not only compromise the patient’s cognitive functions (as part of HAND development) but also affects peripheral organs as a result of chronic and persistent inflammation. Although significant advances have been made regarding HIV-induced pathologies, an effective adjunctive therapy to mitigate chronic inflammation to avoid damage to organs and/or HAND development remains elusive. The inflammation suffered by patients with HIV represents a major clinical challenge because it is necessary to rectify disproportioned inflammation processes in the CNS and periphery simultaneously. An anti-inflammatory medication that crosses the BBB and acts in the periphery could be an ideal strategy to decrease inflammation on the CNS and periphery in patients infected with HIV. Moreover, a pharmacological target expressed in both CNS and periphery represents a unique opportunity to counteract neuroinflammation/peripheral inflammation and to prevent HAND as well as the appearance of non-AIDS-related diseases. Indeed, this is what the α7-nAChR offers: a new therapeutic target for adjunctive therapy expressed in the brain and immune cells. In the brain, α7-nAChR activation improves cognitive processes (Thomsen et al. 2010) and decreases neuroinflammation (Egea et al. 2015), whereas in the periphery, α7-nAChR activation in macrophages and monocytes inhibits the production of proinflammatory cytokines (Wang et al. 2003; Yoshikawa et al. 2006). The inflammatory process that is developed in the brain of patients infected with HIV has been the focus of recent studies. Neuroinflammation occurs when microglia, astrocytes, and perivascular macrophages are activated. Therefore, α7-nAChR clearly represents a unique pharmacological target that could be exploited in the HIV field. Still, it is important to consider that α7-nAChR is also expressed in other immune cells such as lymphocytes and other non-immune cells in which the net effect of its systemic activation, modulation, or blockade is still unknown.

Because the α7-nAChR has received much attention by the pharmaceutical industry, many molecules that target this receptor have recently been developed. According to clinicaltrials.gov (consulted June 18, 2017), eleven trials with different drugs targeting α7-nAChR have been recently executed, including nine completed, one terminated, and one active (recruiting). In addition to the synthesis of new therapeutics, it is also possible to take advantage of FDA-approved drugs available in the market. For instance, the antidepressant and smoking cessation medication bupropion is an FDA-approved α7-nAChR antagonist that has been used in patients infected with HIV (Park-Wyllie and Antoniou 2003; Currier et al. 2003; Pedrol-Clotet et al. 2006). Importantly, in HIV-negative individuals and animal models, bupropion use has demonstrated anti-inflammatory properties (Altschuler and Kast 2003; Brustolim et al. 2006). Furthermore, bupropion has been shown to decrease some chemokines, but not interleukins, in human macrophages upregulated for α7-nAChR by the HIV-gp120_IIIB_ (Delgado-Vélez et al. 2015) (Fig. 1). Indeed, drugs that agonize the α7-nAChR with anti-inflammatory benefits have been described (de Jonge and Ulloa 2007; Pohanka 2012), and new potential compounds continuously emerge (Sahdeo et al. 2014; van Maanen et al. 2015).

Another plausible medication is varenicline, a full agonist of α7-nAChRs (Mihalak et al. 2006) and smoking cessation drug that has been demonstrated to be safe and effective in patients infected with HIV (Cui et al. 2012; Ferketich et al. 2013). Results from varenicline suggest similar or even higher cessation rates than for the general population (Cui et al. 2012; Ferketich et al. 2013; Shirley et al. 2013). Thus, repurposing FDA-approved pharmacotherapeutics targeted at α7-nAChRs to treat HIV-associated diseases implies that new treatments could reach patients faster.

An understudied perspective on HIV-induced chronic inflammation and HAND is the role of nAChRs. Here, we provide evidence demonstrating that the α7-nAChR is upregulated in HIV settings in a gp120-dependent manner in both neural and immune cells (Ballester et al. 2012; Ramos et al. 2015; Delgado-Vélez et al. 2015; Capó-Vélez et al. 2018) and that HIV-infected monocytes, macrophages, and T-cells are also upregulated in HIV-infected women (Delgado-Vélez et al. 2015). The observed increase in α7-nAChR levels is in accordance with the α7-nAChR upregulation reported under inflammation settings in T-lymphocytes, alveolar macrophages, and neutrophils (Su et al. 2007; Chernyavsky et al. 2009; Nizri et al. 2009). Apparently, α7-nAChR upregulation is a typical response of immune cells during inflammation challenges, whereas in the CNS it appears to be a response induced by the HIV viral glycoprotein, gp120 (Ballester et al. 2012; Capó-Vélez et al. 2018) or the viral presence itself. Overall, the α7-nAChR offers the opportunity to explore new pharmacological treatments through the development of new drugs or by testing available drugs for HIV treatment.

It is not clear whether high levels of α7-nAChRs in immune or neuronal cells (particularly microglia and macrophages) represent an anti-inflammatory ally that can be exploited as a therapeutic target since our recent results demonstrate that upregulation of α7-nAChRs does not potentiate the anti-inflammatory response in macrophages (Delgado-Vélez et al. 2015). Moreover, whether the upregulation observed in neural cells and neural tissues (Ballester et al. 2012; Ramos et al. 2015; Capó-Vélez et al. 2018) represents a pharmacological intervention opportunity remains undetermined. However, positive allosteric modulators of α7-nAChRs, such as PNU-120596, can be tested to try to rescue α7-nAChR’s anti-inflammatory activity (Hurst et al. 2005). Thus, efforts to better understand the molecular mechanism that impede the appropriate function of the cholinergic anti-inflammatory response in macrophages in HIV settings are definitely critical in the development of new strategies to reduce neuroinflammation and peripheral inflammation in patients infected with HIV. Moreover, more research is necessary to better understand the role of α7-nAChR in the neurocognitive deterioration experienced by patients infected with HIV.
